# Evaluation of Mass Spectrometry-Based Detection of Panfungal Serum Disaccharide for Diagnosis of Invasive Fungal Infections: Results from a Collaborative Study Involving Six European Clinical Centers

**DOI:** 10.1128/JCM.01867-18

**Published:** 2019-04-26

**Authors:** Marjorie Cornu, Boualem Sendid, Alexandre Mery, Nadine François, Malgorzata Mikulska, Valérie Letscher-Bru, Elena De Carolis, Lauro Damonti, Marie Titecat, Pierre-Yves Bochud, Alexandre Alanio, Maurizio Sanguinetti, Claudio Viscoli, Raoul Herbrecht, Yann Guerardel, Daniel Poulain

**Affiliations:** a Laboratoire de Parasitologie Mycologie, CHU Lille, Université Lille, INSERM U995-LIRIC (Lille Inflammation Research International Centre), Lille, France; b Unité de Glycobiologie Structurale et Fonctionnelle UMR CNRS, Université de Lille 1, Villeneuve d'Ascq, France; c Division of Infectious Disease, Ospedale Policlinico San Martino, Department of Health Sciences, University of Genoa, Genoa, Italy; d Institut de Parasitologie et de Pathologie Tropicale-Université de Strasbourg, Laboratoire de Mycologie Médicale, CHU de Strasbourg, Strasbourg, France; e Department of Laboratory Sciences and Infectious Diseases, Fondazione Policlinico Universitario A. Gemelli IRCCS, Rome, Italy; f Service des Maladies Infectieuses, Centre Hospitalier Universitaire Vaudois, Lausanne, Switzerland; g Laboratoire de Bactériologie CHU Lille, Université Lille, INSERM U995-LIRIC (Lille Inflammation Research International Centre), Lille, France; h Institut Pasteur, Molecular Mycology Unit, Département de Mycologie, CNRS UMR2000, Paris, France; i Laboratoire de Parasitologie-Mycologie, Hôpital Saint-Louis, Groupe Hospitalier Lariboisière, Saint-Louis, Fernand Widal, Assistance Publique-Hôpitaux de Paris (AP-HP), Paris, France; j Université Paris Diderot, Sorbonne Paris Cité, Paris, France; k Oncologie et Hématologie, INSERM, UMR-S1113/IRFAC, Hôpitaux Universitaires de Strasbourg et Université de Strasbourg, Strasbourg, France

**Keywords:** invasive aspergillosis, invasive candidiasis, invasive fungal infection, mass spectrometry, mucormycosis, serological diagnosis

## Abstract

A mass spectrometry (MS) method that detects a serum disaccharide (DS) (MS-DS) was recently described for the diagnosis of invasive fungal infections (IFI). We carried out a European collaborative study to evaluate this assay. Patients with the following IFI were selected according to the availability of sera obtained at about the time that IFI was documented: invasive candidiasis (IC; *n* = 26 patients), invasive aspergillosis (IA; *n* = 19), and mucormycosis (MM; *n* = 23). Control sera originated from 20 neutropenic patients and 20 patients with bacteremia. MS-DS was carried out in blind manner for the diagnosis of IFI. A diagnosis of IC or IA was confirmed by detection of mannan (Man) or galactomannan (GM), respectively, associated with detection of (1,3)-β-d-glucan (BDG) in both infections. MM was detected by quantitative real-time PCR (qPCR). All tests discriminated sera from patients with IC from sera from control subjects with bacteremia (*P* ≤ 0.0009). For IC, the MS-DS sensitivity and specificity were 51% and 87%, respectively. MS-DS complemented the high specificity of Man monitoring. All tests discriminated sera from IA patients from sera from neutropenic controls (*P* ≤ 0.0009). For IA, MS-DS sensitivity and specificity were 64% and 95%, respectively. Only 13/36 serum samples from patients with MM were concordant by MS-DS and qPCR (6 were positive, and 7 were negative); 14 were positive by MS-DS alone. qPCR and MS-DS made a similar contribution to the diagnosis of MM. In patients undergoing long-term monitoring, the persistent circulation of serum disaccharide was observed, whereas DNA was detected only for a short period after initiation of treatment. MS-DS has an important role to play in the early diagnosis of IFI. Its panfungal nature and complementarity with other tests may justify its use in the management of IFI.

## Abstract (precis)

A mass spectrometry (MS) method that detects a serum disaccharide (DS) (MS-DS) was recently described for the diagnosis of invasive fungal infections (IFI). We carried out a European collaborative study to evaluate this assay.

## INTRODUCTION

Numerous reports have described the increasing problem of nosocomial invasive fungal infections (IFI) in immunocompromised patients (1, 2). This is due to the increased number of at-risk patients in parallel with progress in intensive care and/or hematology, leading to deeper or longer immunosuppression (3). In parallel, the spectrum of isolated fungi has shifted from well-known opportunistic pathogens with characterized virulence factors (4, 5) to other species rarely reported to be a cause of human infection (6–8). Disruptions in the microbial balance induced by antibacterial antibiotics favor fungal fitness (9). Antifungal therapy is generally prescribed in patients with persistent fever despite 3 days of antibiotic treatment (10). Diagnostic strategies involve conventional methods of isolation and identification of fungi from blood or sterile sites, imaging, and a panel of biological tests whose nature depends on the fungal agent suspected (11, 12). The few biological tests currently considered by physicians to be of diagnostic help do not involve changes in the host response related to fungal infections (e.g., antifungal antibodies [13] or cytokine profiling [14]) but consist of the detection of circulating fungal molecules, either glycans or DNA, in the patients' sera using tests developed with the advent of hybridoma technology (15, 16), discoveries in biochemical cascades (17), or PCR (18). Due to the poor sensitivity of conventional mycological methods (12, 19), infectious disease societies have produced different levels of recommendations for immunological tests for the diagnosis of invasive candidiasis (IC) and invasive aspergillosis (IA) (20, 21). These tests, which are considered specific but which sometimes lack sensitivity, were later complemented by recommendations for the more sensitive Fungitell test, which detects both IC and IA (22). Despite years of extensive research into the detection of fungal DNA, a consensus has been reached only for IA, while standardization is still in progress for IC and mucormycosis (MM) (23). For MM, a standardized PCR method would be of primary interest since, in contrast to IC, glycan detection tests are not available (24). Recently, the T2MR technology, combining DNA amplification and detection by magnetic resonance, has provided significant progress in terms of reducing the delay in the time to diagnosis in comparison with the time to diagnosis by blood cultures for IC (25). More recently, matrix-assisted laser desorption ionization–time of flight (MALDI-TOF) mass spectrometry (MS) has become essential in clinical mycology laboratories, providing a more rapid and accurate means of identification of fungal species isolated from patients (26).

We developed an approach based on the ability of MS to detect fungal molecules in patients’ sera (27). Subsequent work showed that this method, which allows the detection and relative quantification of a panfungal serum disaccharide (DS), gave results that were comparable to those of methods recommended for the diagnosis of IFI (28). Here, we describe the results of a European collaborative study carried out in a blind manner to validate the use of MS for DS (MS-DS) for the diagnosis of IFI.

(This study was presented in part at the Russian Scientific Conference on Medical Microbiology and Clinical Mycology, Saint Petersburg, Russia, 14 to 16 June 2016; at the 13th Annual Fungal Update meeting, St Bartholomew's Hospital, London, United Kingdom, 2 to 3 March 2018; and as an e-poster at the 20th Congress of the International Society for Human and Animal Mycology, Amsterdam, Netherlands, 30 June to 4 July 2018 [29].)

## MATERIALS AND METHODS

### Study design and participants.

Different centers provided sera from patients with IFI classified as proven or probable according to the European Organization for the Research and Treatment of Cancer (EORTC)/Mycoses Study Group (MSG) criteria (30). Patients with IC were selected at the University Hospital of Genoa and Catholic University of Sacred Heart, Rome, Italy. Controls for IC patients consisted of febrile patients who were admitted to the Emergency Department of the University Hospital of Lausanne, Lausanne, Switzerland, and in whom bacteremia was subsequently documented. Two cases of nocardiosis from Lille University Hospital were also included among the controls. Patients with IA were selected at the University Hospital of Strasbourg, Strasbourg, France. Controls consisted of neutropenic patients hospitalized at the University Hospital of Genoa. Patients with MM were selected at Saint Louis University Hospital, Paris, France, and Lille University Hospital, Lille, France.

Altogether 95 patients and 189 serum and plasma samples were selected. They were distributed as follows: 17 patients with IC (27 serum samples), 19 patients with IA (53 serum samples), 16 patients with MM (36 serum samples), 10 control neutropenic patients (20 serum samples), 20 patients (21 plasma samples) with bacterial infections, and 2 patients with *Actinomycete* infections (2 serum samples).

### MS-DS detection.

Serum and plasma samples were frozen and sent on dry ice to the Laboratory of Clinical Mycology, Lille University Hospital, for processing as described previously (27). After a preanalytical step for the extraction and purification of oligosaccharides, spectra were recorded and analyzed using a 4800 MALDI-TOF/TOF analyzer (Applied Biosystems/MDS Sciex) at a fixed laser intensity within a 300- to 800-*m/z* range (UMR CNRS 8576, University of Lille).

### Detection of circulating poly- and oligosaccharides and DNA in serum or plasma.

For the diagnosis of IC and IA, detection of mannan (Man) or galactomannan (GM) and (1,3)-β-d-glucan (BDG) was performed in the participating centers during routine patient screening or in the Lille Clinical Mycology Laboratory if they had not been tested previously.

BDG was measured using a Fungitell kit (Associates of Cape Cod Inc., Falmouth, MA, USA) following the manufacturer’s instructions. The recommended cutoff of 80 pg/ml was used to determine clinical relevance.

Measurement of serum Man and GM was performed using a Platelia Candida Ag Plus test and Platelia Aspergillus Ag test (Bio-Rad, Marnes la Coquette, France), respectively, according to the manufacturer's instructions. The recommended cutoffs of a concentration of 62.5 pg/ml and an index of ≥0.5, respectively, were used.

For the diagnosis of MM, quantitative real-time PCR (qPCR) was performed as described previously (24, 31).

### Ethics statement.

No additional sampling was necessary in any center due to the retrospective nature of the study. In Lille, agreement for the establishment of a biological collection of IFI samples was obtained from the French Ministry of Education and Research under reference number DC2008-642. Institutional review board approval was granted by the Comité de Protection des Personnes Nord-Ouest IV, the ethical committee of the university hospital of Lille.

### Statistical analysis.

GraphPad Prism (version 6) software was used to compare the distribution of biomarkers in the different groups with the Mann-Whitney two-tailed test and to generate receiver operating characteristic (ROC) curves, derive cutoffs, and construct graphs. A *P* value of <0.05 was considered statistically significant.

## RESULTS

### Invasive candidiasis.

**(i) Study population.** The origin of the IC patients, the delay to serum sampling in relation to the time of the first positive blood culture, and the *Candida* species isolated are shown in Table 1. The *Candida* species were representative of the usual epidemiology encountered in southern Europe, with a higher prevalence of Candida parapsilosis complex isolates. The control patients with bacteremia are listed in Table 2. These included the usual panel of patients with community-acquired bacterial infections and two cases of *Nocardia* infection.

**TABLE 1:** Origin of sera from patients with IC, delay between serum sampling and time of first positive blood culture, and *Candida* species isolated^*a*^

Patient no. (site)	Species isolated	Delay vs BC (days)	Man concn (pg/ml)	BDG concn (pg/ml)	MS-DS index
I1 (G)	Candida albicans	2	**>500**	**688**	**2,000**
	C. albicans	5	**>500**	**1,528**	**550**
	C. albicans	7	**>500**	**861**	**625**
I2 (G)	C. albicans	2	**81**	**943**	79
	C. albicans	5	**192**	**405**	133
	C. albicans	7	**79**	**286**	200
I3 (G)	C. albicans	2	**69**	**155**	**400**
	C. albicans	7	30	**108**	**526**
	C. albicans	13	12	50	300
I4 (G)	C. albicans	0	**>500**	**5,000**	**1,000**
	C. albicans	5	**>500**	**5,000**	**333**
	C. albicans	7	**>500**	**5,000**	285
I5 (G)	C. albicans	1	**441**	**407**	133
	C. albicans	6	22	**222**	110
	C. albicans	10	38	**336**	300
I6 (G)	C. albicans	−5	**108**	**403**	70
	C. albicans	0	**150**	**283**	85
	C. albicans	4	**232**	**2,528**	500
I7 (G)	C. albicans	2	**>500**	**850**	350
	C. albicans	10	**>500**	**367**	667
I8 (G)	C. albicans	1	41	**158**	238
	C. albicans	7	39	58	151
I9 (R)	C. albicans	1	4	**>500**	**1,000**
I10 (R)	C. albicans	2	0	**>500**	**385**
I11 (R)	C. albicans	1	3	**>500**	175
	C. albicans	0	3	**>500**	**435**
I12 (R)	C. albicans	1	**>500**	**>500**	159
I13 (R)	C. albicans	1	0	**>500**	122
I14 (R)	C. glabrata	0	**64**	**>500**	78
I15 (G)	C. tropicalis	3	**>500**	**159**	110
	C. tropicalis	7	18	**189**	**357**
	C. tropicalis	14	31	42	**345**
I16 (R)	C. tropicalis	3	>500	>500	**714**
I17 (G)	C. parapsilosis	2	5	61	53
	C. parapsilosis	5	0	**245**	172
	C. parapsilosis	6	**82**	7	238
I18 (G)	C. parapsilosis	1	16	**125**	122
	C. parapsilosis	4	14	**159**	**400**
	C. parapsilosis	14	34	70	**667**
I19 (G)	C. parapsilosis	0	21	26	159
	C. parapsilosis	4	18	77	65
I20 (G)	C. parapsilosis	−1	**110**	39	**833**
	C. parapsilosis	3	6	20	**450**
I21 (R)	C. parapsilosis	1	2	**>500**	**1,000**
I22 (R)	C. parapsilosis	1	NA	**>500**	109
	C. parapsilosis	1	NA	**>500**	106
I23 (R)	C. parapsilosis	7	**256**	**>500**	333
I24 (R)	C. parapsilosis	0	0	**>500**	96
I25 (R)	C. orthopsilosis	1	**>500**	**>500**	769
	C. orthopsilosis	1	**>500**	**>500**	588
	C. orthopsilosis	1	**>500**	**>500**	**1,000**
	C. orthopsilosis	1	**>500**	**>500**	**625**
I26 (G)	C. krusei	3	12	**327**	**357**
	C. krusei	7	10	**385**	**344**
	C. krusei	10	8	**191**	**400**
I27 (G)	C. krusei	2	12	7	300
	C. krusei	4	16	14	167

a BC, time of first positive blood culture; BDG, (1,3)-β-d-glucan; G, Genoa, Italy; Man, mannan; MS-DS, mass spectrometry method for detection of a serum disaccharide; NA, not available; R, Rome, Italy. Bold characters correspond to positive values.

**TABLE 2:** Origin of the control sera used in the MS-DS test for invasive candidiasis^*a*^

Patient no. (site)	Reason(s) for hospitalization	Risk factor(s) for fungal infection	Species isolated	Delay vs BC (days)	Man concn (pg/ml)	BDG concn (pg/ml)	MS-DS index
S1	Septic shock, infected kidney stone	Corticosteroids, panhypopituitarism (substitution)	Escherichia coli	−1	2	**97**	230
S2	Septic shock, urinary infection	MGUS, CRF, corticosteroids (unconfirmed Horton’s disease)	E. coli	0	3	**83**	**286**
S3	Cholangitis	Corticosteroids, polymyalgia rheumatica	E. coli	0	4	7	70
S4	Urosepsis	IDDM, PPI	E. coli	0	1	7	65
S5	Urosepsis	CRF, chronic ulcers, gout	E. coli	0	9	**169**	**400**
S6	Cholecystitis	PPI	E. coli	0	3	29	122
S7	Urosepsis	CRF, corticosteroids, polymyalgia rheumatica, PPI	E. coli	0	0	7	**667**
S8	Urosepsis	Indwelling urinary catheter	E. coli	0	0	**122**	43
S9	Skin infection	CRF	Streptococcus agalactiae	0	0	7	120
S10	Skin infection	None	Streptococcus dysgalactiae	0	2	7	154
S11	Skin infection	None	S. dysgalactiae	0	1	22	76
S12	Pneumococcal pneumonia	NIDDM	Streptococcus pneumoniae	0	3	7	55
S13	Pneumonia	None	Streptococcus pyogenes	0	4	7	**500**
S14	Plantar abscess	IDDM, metabolic syndrome	S. pyogenes	0	0	7	115
S15	Wound infection	IDDM, diffuse vascular disease, PPI	Staphylococcus aureus	0	4	**280**	238
S16	Endocarditis/spondylodiscitis	PPI	S. aureus	4	2	7	53
S17	Pyelonephritis	IDDM	Klebsiella pneumoniae	1	7	7	54
S18	Urosepsis	Indwelling urinary catheter	Proteus mirabilis	2	3	77	65
S19	Stroke, dental abscess	Diffuse cerebrovascular disease, PPI	Parvimonas micra	0	1	7	106
S20	Skin infection	IDDM, diffuse vascular disease	*Corynebacterium* spp.	0	4	7	104
S21	Cholangitis	None	Enterococcus faecalis, Enterobacter cloacae, Pseudomonas aeruginosa	0	2	7	115
L1	Pneumonia	Renal graft and leukemia	Nocardia nova	−2	0	35	72
L2	Skin infection and adenitis	Renal graft	N. nova	−5	0	**2,336**	**131**

a BC, time of first positive blood culture; BDG, (1,3)-β-d-glucan; CRF, chronic renal failure; IDDM, insulin-dependent diabetes mellitus; L, Lille, France; Man, mannan; MGUS, monoclonal gammopathy of undetermined significance; MS-DS, mass spectrometry method for detection of a serum disaccharide; NNIDDM, non-insulin-dependent diabetes mellitus; PPI, proton pump inhibitor; S, Switzerland. Bold characters correspond to positive values.

**FIG 1:**
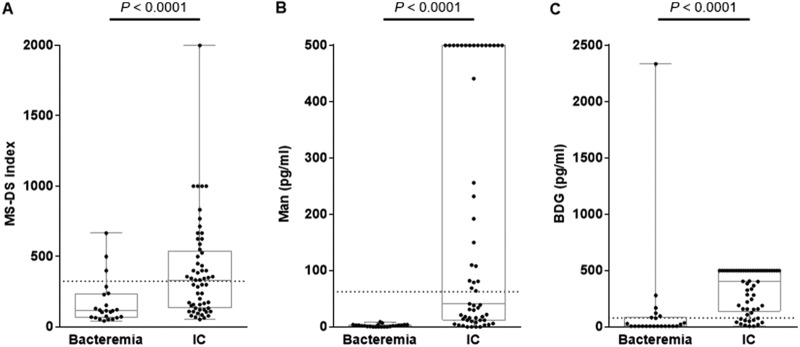
Distribution of MS-DS indexes (A), Man concentrations (B), and BDG concentrations (C) in patients with invasive candidiasis (IC) versus the controls. The results for each biomarker for the patient and control groups were compared using the Mann-Whitney test (significant at *P* ≤ 0.05). The dotted lines represent the cutoff values for each biomarker.

**(ii) MS-DS diagnosis of IC in comparison to BDG and Man detection.** The distribution of BDG and Man concentrations and MS-DS index values is shown in Fig. 1. All tests significantly discriminated IC patients from bacteremic controls (*P* < 0.0001).

Among the 27 IC patients, 9 were positive by all three tests, 12 were positive by two tests (6 by BDG detection and MS-DS; 5 by BDG and Man detection; 1 by Man detection and MS-DS), and 4 were positive by BDG detection alone. Only two patients (patients I19 and I27) infected by C. parapsilosis and C. krusei were negative by all tests. When considering control patients with bacteremia, three were positive by MS-DS, whereas six were positive for BDG, including the patient with *Nocardia* infection who displayed very high glucan levels. Only one control (patient S5) was positive for two biomarkers (BDG and DS).

**FIG 2:**
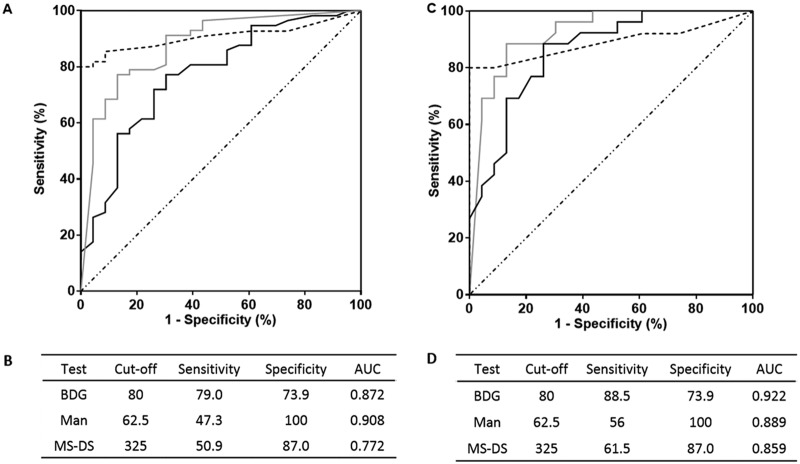
(A, C) ROC curves for serum (A) and patients (C) for invasive candidiasis. Gray, dashed, and black lines, results for BDG detection, Man detection, and MS-DS, respectively. (B, D) Sensitivity/specificity values according to preestablished cutoff values for each biomarker for analysis of serum (B) and patients (D). AUC, area under the concentration-time curve.

**FIG 3:**
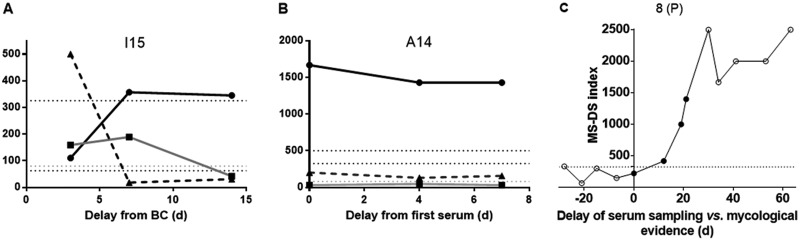
Examples of kinetics of serum biomarkers in patients with invasive candidiasis (IC) (A), invasive aspergillosis (IA) (B), and mucormycosis (MM) (C). Day 0 indicates the date of a positive fungal blood culture (BC) for IC or the first available serum sample for IA and the date of mycological evidence for MM. (A, B) Black circles and black solid line, MS-DS; black triangles and dashed line, Man or GM detection; black squares and gray line, BDG detection. Biomarker levels are indicated on the *y* axis with reference to BDG values (pg/ml), MS-DS index, and GM (index × 1,000). (C) Empty and full circles, negative and positive Mucorales qPCR results, respectively, for MM patients. Horizontal dotted lines indicate the cutoff values for BDG (80 pg/ml), Man (62.5 pg/ml), GM (index value, 0.5 · 1,000), and MS-DS (index value, 325). d, day.

Figure 2 shows the ROC curves and corresponding sensitivities and specificities for the MS-DS, BDG, and Man detection tests. When considering the results for serum samples (Fig. 2A and B), application of the cutoff value of 325 for MS-DS showed a sensitivity of 51% and a specificity of 87%, which were intermediate values compared to those obtained by the BDG and Man tests. Analysis of the MS-DS index values for IC diagnosis was then performed for patients (Fig. 2C and D), and the sensitivity reached 67% without altering the high specificity estimated for serum. Comparison of ROC curves established for the MS-DS and the BDG and Man tests revealed that the diagnostic value of MS-DS was similar to that of the BDG test and positively complemented the high specificity of Man monitoring (revealed by the asymptotic curve). A lack of concordance between MS-DS and the BDG and Man tests was observed for serial serum samples from a given patient. This appeared to be more moderate when considering the global biomarker patterns per patient, since most patients (22/26) aggregated into two groups: those that displayed three positive test and those that displayed two positive tests. Figure 3A shows an example of biomarker kinetics during the time course of IC in one patient. Tests for BDG and Man were positive on day 1 and decreased on day 7, whereas MS-DS became positive at the end of monitoring. Due to the retrospective nature of the study, few serum samples were available per patient, but analysis of the whole IC patient population confirmed the transient nature of Man detection, in contrast to a slower decrease in glucanemia.

### Invasive aspergillosis.

**(i) Study population.** The characteristics of the IA patients, the level of certainty of a diagnosis of IA according to EORTC criteria, and the *Aspergillus* species isolated are shown in Table 3. Except for one patient with invasive sinusitis, all patients presented with invasive pulmonary aspergillosis. “Day 0” indicates the date of the first serum available in the collection relative to the episodes of IA defined according to clinical and radiological arguments.

**TABLE 3:** Origins of sera from patients with invasive aspergillosis, *Aspergillus* species isolated, and level of evidence of *Aspergillus* infection^*a*^

Patient no.	Age (yr)	Sex	Underlying condition(s)	Species isolated	EORTC classification	Treatment	Outcome	GM in BAL fluid	Delay between serum sample collections (days)	BDG concn (pg/ml)	GM index in serum	MS-DS index
A1	44	M	Heart transplant	Aspergillus fumigatus	Proven IPA	VCZ	Complete remission, alive at wk 12	NA	0	**4,279**	**>6**	**350**
									7	**5,000**	**4.06**	**333**
									17	**4,362**	**2.63**	**555**
									24	**4,045**	**2.12**	**333**
A2	56	M	CLL with Richter transformation	NA	Probable IPA	VCZ	Progression, death on day 28	Pos	0	**134**	**1.09**	123
									11	42	0.17	**400**
									16	**98**	0.16	**714**
A3	90	M	AML	A. fumigatus	Probable IPA	VCZ	Progression, death on day 44	NA	0	7	0.09	113
A4	63	M	COPD, corticosteroids, diabetes mellitus	A. fumigatus	Probable IPA	VCZ	Complete remission, alive at wk 12	NA	0	**1,643**	**1.59**	58
A5	35	M	Lymphoma, MAS	NA	Probable IPA	VCZ	Progression, death on day 66	NA	0	60	**0.97**	90
									3	79	**0.55**	76
A6	56	F	Liver transplant	A. fumigatus	Probable IPA	VCZ	Complete remission, alive at wk 12	NA	0	**489**	**2.30**	**3,333**
									8	**235**	**0.87**	**1,250**
									15	**340**	**0.57**	**2,000**
A7	59	M	Liver transplant	Aspergillus nidulans	Probable IPA	VCZ and then VCZ + CAS	Progression, death on day 84	Pos	0	40	**1.02**	**526**
									10	78	**0.54**	**400**
									21	64	0.34	**526**
A8	56	M	ALL	NA	Probable IPA	VCZ	Progression, alive at wk 12	Pos	0	7	0.06	105
									10	7	0.05	135
									19	35	0.05	72
A9	32	M	Testicular cancer	NA	Probable IPA	VCZ and then L-AMB	Progression, death on day 71	Pos	0	73	**1.37**	**1,250**
									4	**102**	**0.67**	**1,667**
									10	66	0.35	**3,333**
A10	53	F	AML, Allo-HSCT	A. fumigatus	Proven IA sinusitis	L-AMB	Partial response, death on day 61	NA	0	7	**0.55**	87
									3	36	**0.98**	**625**
									9	7	0.22	**1,000**
A11	54	M	Heart and liver transplant	A. fumigatus	Probable IPA	VCZ and then L-AMB	Progression, death on day 82	Pos	0	**949**	0.44	**714**
									14	**1,999**	0.51	**333**
									21	**1,776**	0.31	**833**
A12	26	M	Lymphoma, HTLV-1, Allo-HSCT	NA	Probable IPA	VCZ	Stable, alive at wk 12	NA	0	**208**	3.32	**1,429**
									3	**454**	**0.56**	**5,000**
									14	**312**	0.31	**1,429**
A13	62	M	CMML, COPD	A. fumigatus	Probable IPA	VCZ and then VCZ + CAS	Partial response, alive at wk 12	Pos	0	**238**	0.13	127
									11	**195**	0.07	78
									15	**217**	0.06	83
A14	47	M	Posthepatitis liver fibrosis	A. fumigatus	Probable IPA	L-AMB	Progression, death on day 27	Pos	0	31	0.20	**1,667**
									4	48	0.13	**1,429**
									7	30	0.16	**1,429**
A15	71	M	Multiple myeloma	NA	Probable IPA	VCZ	Progression, death on day 54	Pos	0	7	0.08	**333**
									3	7	0.08	**400**
									9	7	0.07	300
A16	60	M	AML	NA	Probable IPA	VCZ	Progression, death on day 21	Pos	0	27	**2.39**	149
									7	7	**1.89**	52
A17	51	M	Lymphoma	NA	Probable IPA	VCZ and then L-AMB	Complete response, alive at wk 12	NA	0	12	**0.73**	61
									4	7	0.11	109
									6	20	0.07	238
A18	73	M	CLL	NA	Probable IPA	VCZ	Progression, death on day 31	NA	0	73	**0.82**	109
									11	**292**	**>6**	**588**
									24	**111**	**3.08**	**625**
A19	39	F	Lymphoma	NA	Probable IPA	VCZ and then L-AMB	Progression, death on day 61	NA	0	**3,030**	**>6**	**714**
									8	**517**	**3.34**	**2,000**
									15	**479**	**1.22**	**3,333**

a ALL, acute lymphoblastic leukemia; Allo-HSCT, allogeneic hematopoietic stem cell transplantation; AML, acute myeloid leukemia; BAL, bronchoalveolar lavage; BDG, (1,3)-β-d-glucan; CAS, caspofungin; CLL, chronic lymphoblastic leukemia; CMML, chronic myelomonocytic leukemia; COPD, chronic obstructive pulmonary disease; EORTC, European Organization for Research and Treatment of Cancer; F, female; GM, galactomannan; HTLV-1, human T cell leukemia virus type 1; IPA, invasive pulmonary aspergillosis; L-AMB, liposomal amphotericin B; M, male; MAS, macrophage activation syndrome; MS-DS, mass spectrometry method for detection of a serum disaccharide; NA, not available; Pos, positive; VCZ, voriconazole. Bold characters correspond to positive values.

The characteristics of the controls, consisting of neutropenic patients, are summarized in Table 4. Retrospective analysis of the clinical evolution of IA revealed that control patients 3, 6, and 7 developed probable IA 1, 2, and 6 months after serum sampling, respectively.

**TABLE 4:** Origin of control sera used in MS-DS test for invasive aspergillosis^*a*^

Patient no.	Age (yr)	Sex	Underlying condition(s)	Antifungal prophylaxis	Delay between serum sample collections (days)	BDG concn (pg/ml)	GM index in serum	MS-DS index
1	50	M	Neutropenia, myelofibrosis	Fluconazole	0	7	0.07	68
					3	7	0.08	86
2	40	M	Neutropenia, Hodgkin’s disease	Fluconazole	0	7	0.09	147
					4	7	0.06	143
3	48	F	Neutropenia, Allo-HSCT (ALL)	No	0	7	0.18	200
					5	7	0.21	81
4	44	F	Neutropenia/GVHD, Allo-HSCT (myelofibrosis)	Posaconazole	0	7	0.04	161
					1	7	0.04	120
5	21	F	Neutropenia, Allo-HSCT (AML)	Posaconazole	0	7	0.05	66
					7	7	0.05	39
6	49	M	Neutropenia, Allo-HSCT (myeloma)	Fluconazole	0	7	0.10	**400**
					3	7	0.06	200
7	57	F	Neutropenia/GVHD, Allo-HSCT (AML)	Fluconazole	0	7	0.03	200
					7	7	0.04	**300**
8	75	F	Neutropenia, AML	Fluconazole	0	41	0.03	85
					3	7	0.03	49
9	36	M	Neutropenia, AML	Posaconazole	0	7	0.05	58
					3	7	0.03	86
10	52	F	Neutropenia, AML	Posaconazole	0	7	0.05	50
					11	7	0.05	51

a ALL, acute lymphoblastic leukemia; Allo-HSCT, allogeneic hematopoietic stem cell transplantation; AML, acute myeloid leukemia; BDG, (1,3)-β-d-glucan; F, female; GM, galactomannan; GVHD, graft-versus-host disease; M, male; MS-DS, mass spectrometry method for detection of a serum disaccharide. Bold characters correspond to positive values.

**FIG 4:**
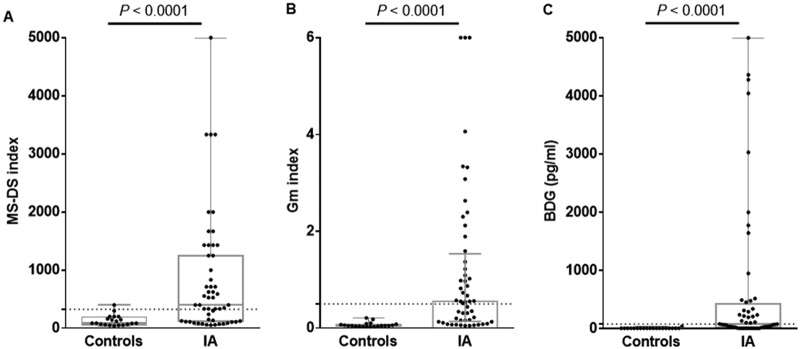
Distribution of MS-DS indexes (A), GM indexes (B), and BDG concentrations (C) in patients with invasive aspergillosis (IA) and controls. The patient and control groups were compared using the Mann-Whitney test (significant at *P* ≤ 0.05). The dotted lines represent the cutoff values for each biomarker.

**(ii) MS-DS diagnosis of IA in comparison to BDG and GM detection.** The distribution of BDG concentrations and MS-DS index values is shown in Fig. 4. The values for the biomarkers were significantly higher in IA patients than in the controls (*P* ≤ 0.0001).

**FIG 5:**
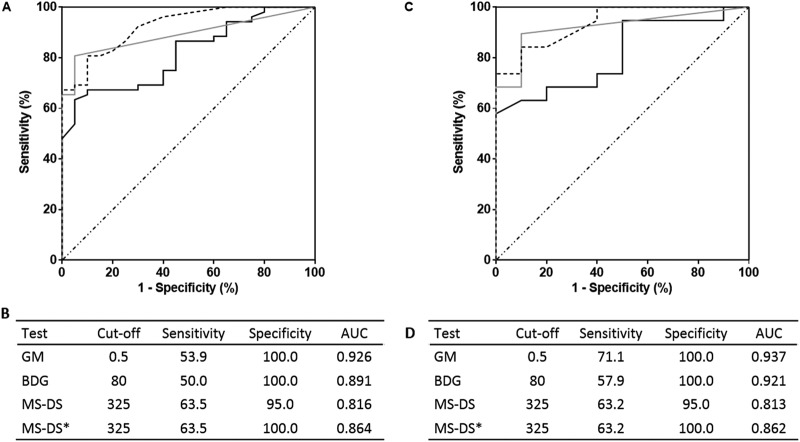
(A, C) ROC curves for serum (A) and patients (C) for invasive aspergillosis (IA). (B, D) Sensitivity/specificity values according to preestablished cutoff values for each biomarker for analysis of serum (B) and patients (D). MS-DS*, results obtained by exclusion of the control who developed IA 2 months later. Gray, dashed, and black lines, results for BDG detection, GM detection, and MS-DS, respectively.

The sensitivity, specificity, and cutoff values for MS-DS and BDG detection were assessed by establishing ROC curves for serum, as shown in Fig. 5A and B. With a cutoff value of 325 for the MS-DS index, the sensitivity and specificity were 64% and 95%, respectively. The sensitivity values were intermediate between those for GM and BDG detection, which had 100% specificity. Interestingly, the 95% specificity of MS-DS was due to the high MS-DS index observed for control patient 6, who subsequently developed IA. In Fig. 5C and D, an analysis of MS-DS index values for IA diagnosis was performed for the patients, and no difference in terms of sensitivity and specificity was revealed with the data obtained for serum. A comparison of MS-DS and BDG detection ROC curves (according to the manufacturer’s recommended threshold) showed that the diagnostic value of MS-DS was better than that of BDG detection, with a maximum sensitivity of 64% for MS-DS and 50% for BDG detection.

Figure 3B shows an example of MS-DS and BDG detection kinetic evolution during GM monitoring. For this patient, only one serum sample was positive at the GM cutoff 14 days after the beginning of monitoring, while BDG levels were already positive on day 0, before increasing at unusually high levels. MS-DS was constantly positive during the whole survey, although it decreased at the time when BDG levels and GM index values were maximum.

### Mucormycosis.

**(i) Study population.** The characteristics of patients and the level of certainty of a diagnosis of MM according to EORTC criteria are shown in Table 5. The genera/species involved were representative of the usual spectra of Mucorales isolated, as were the risk factors, infection sites, and high mortality.

**TABLE 5:** Origin of the sera used in the MS-DS test for detection of mucormycosis^*a*^

Patient no. (site)	Age (yr)	Sex	Underlying condition(s)	Site(s) of MM	Imaging result	Diagnostic sample	Histology/DE	Species	MM level of certainty, EORTC	Treatment	Outcome	Delay vs time of mycological evidence (days)	*C_q_* value by PCR for Mucorales	BDG concn (pg/ml)	GM index in serum	MS-DS index
1 (P)	27	M	Lymphoma	Lung	Bronchiectasis, ground glass	Sputum	NA	*Syncephalastrum* sp. culture	Probable	L-AMB	Death	−15	Neg	NA	<0.5	3,333
												0	Neg	NA	<0.5	123
												7	Neg	NA	<0.5	278
2 (P)	23	F	AML	Lung	Nodule	Biopsy	Hyphae	Lichtheimia corymbifera PCR	Proven	L-AMB	Death	−3	**40**	NA	<0.5	37
3 (P)	18	F	ALL	Liver	Nodule	Biopsy	Hyphae	*Mucor* sp. culture	Proven	L-AMB	Alive	45	Neg	NA	<0.5	**3,333**
												51	Neg	NA	<0.5	**3,333**
4 (P)	75	M	Myelodysplasia	Rhinocerebral	Sinus, eye, and brain invasion	Conjunctival swab	Hyphae	*Lichtheimia* sp. culture	Proven	No	Death	−18	Neg	NA	<0.5	213
												−14	**40**	NA	<0.5	122
												−7	**37**	NA	<0.5	139
5 (P)	72	M	AML	Lung	Nodule	Serum	NA	*Rhizomucor* sp. PCR	Possible	L-AMB	Death	0	**31**	NA	<0.5	213
6 (P)	77	F	ALL	Lung	Nodule	Serum	NA	*Mucor* sp. PCR	Possible	L-AMB	Alive	30	**39**	NA	<0.5	100
7 (P)	19	F	Aplastic Fanconi anemia	Lung and kidney	Nodule	Kidney biopsy	Hyphae	*Rhizomucor* sp. PCR	Proven	L-AMB	Death	45	**39**	NA	<0.5	123
8 (P)	57	M	AML	Liver	Nodule	Biopsy	Hyphae	*Lichtheimia* sp. culture	Proven	L-AMB	Death	−28	Neg	NA	<0.5	**333**
												−21	Neg	NA	<0.5	66
												−15	Neg	NA	<0.5	300
												−7	Neg	NA	<0.5	149
												0	**35**	NA	<0.5	222
												12	**31**	NA	<0.5	**417**
												19	**35**	NA	<0.5	**1,000**
												21	**39**	NA	<0.5	**1,400**
												30	Neg	NA	<0.5	**2,500**
												34	Neg	NA	<0.5	**1,667**
												41	Neg	NA	<0.5	**2,000**
												53	Neg	NA	<0.5	**2,000**
												63	Neg	NA	<0.5	**2,500**
9 (L)	61	F	Allo-HSCT	Lung	Nodule	Biopsy	Hyphae	Rhizopus microsporus culture	Proven	No	Death	−4	**35**	0	0.07	132
10 (L)	66	F	Burns, CML	Skin	NA	Swab	Neg	Lichtheimia ramosa culture	Possible	NS	Alive	−2	Neg	31	NA	132
												5	**37**	18	NA	159
11 (L)	45	F	Allo-HSCT GVHD	Postoperative abscess, abdominal wall (biopsy)	NA	Biopsy	Hyphae	Rhizopus arrhizus culture	Proven	Surgery, hyperbaric oxygen	Death	−9	**33**	42	0.15	300
												6	**34**	46	NA	**2,000**
12 (L)	42	M	Burns	Skin	NA	Biopsy	Hyphae	L. corymbifera culture	Proven	L-AMB, surgery	Alive	24	Neg	21	NA	**1,429**
13 (L)	83	F	Lymphoma, rituximab, diabetes	Lung	Nodule	BAL fluid	Neg	R. microsporus culture	Probable	No	Death	−6	**35**	18	0.06	**455**
												0	**35**	0	0.04	**400**
14 (L)	76	M	Trauma	Skin	NA	Biopsy	Hyphae	Mucor circinelloides culture	Proven	Switch from POSA to L-AMB, surgery, hyperbaric oxygen	Alive	16	Neg	39	NA	**3,333**
15 (L)	60	M	Trauma	Skin	NA	Biopsy	Hyphae	M. circinelloides culture	Proven	L-AMB, surgery	Alive	5	Neg	**106**	0.06	**3,333**
16 (L)	3	F	ALL	Disseminated	Disseminated	Vitreous humor	Hyphae	*Lichtheimia* sp. PCR	Proven	ISA/L-AMB	Alive	−1	Neg	18	0.05	**333**

a ALL, acute lymphoblastic leukemia; Allo-HSCT, allogeneic hematopoietic stem cell transplantation; AML, acute myeloid leukemia; BAL, bronchoalveolar lavage; BDG, (1,3)-β-d-glucan; CML, chronic myelogenous leukemia; *C_q_*, quantification cycle; DE, direct examination; EORTC, European Organization for Research and Treatment of Cancer; F, female; GM, galactomannan; GVHD, graft-versus-host disease; ISA, isavuconazole; L-AMB, liposomal amphotericin B; L, Lille, France; M, male; MM, mucormycosis; MS-DS, mass spectrometry method for detection of a serum disaccharide; NA, not available; Neg, negative; NS, not specified; P, Paris, France; POSA, posaconazole. Bold characters correspond to positive values.

**(ii) MS-DS in comparison with qPCR for diagnosis of MM.** In contrast to IC and IA, no biochemical or immunological assays for the detection of circulating fungal poly- or oligosaccharides are available for the diagnosis of MM. We therefore compared MS-DS with qPCR, which is the only test currently available for the diagnosis of MM. The qPCR method used detects species from the genera *Mucor*, *Rhizomucor*, *Lichtheimia*, and *Rhizopus*.

We tested 36 serum samples from 16 patients (1 to 13 serum samples/patient) by MS-DS and qPCR. The results for only 13 serum samples were concordant by both methods (positive or negative). Among the 23 serum samples with discordant results, 13 were positive by MS-DS only and 10 were positive by qPCR only. Only three patients were positive by both tests. Remarkably, all the patients in this series could be diagnosed with MM by at least one test.

**FIG 6:**
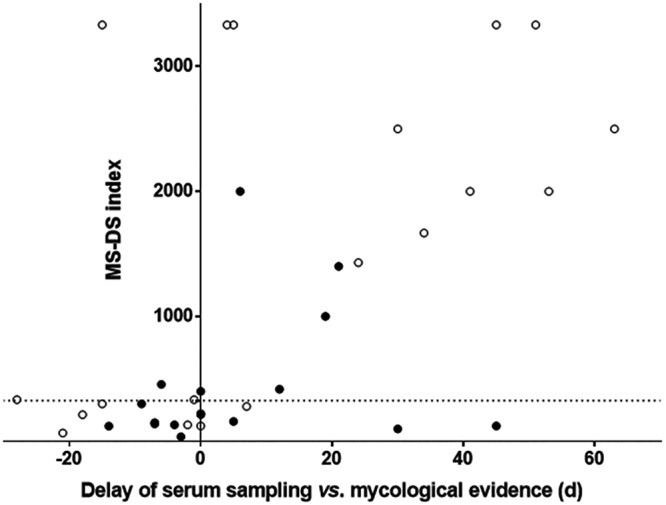
Distribution of DS in relation to the day of mycological diagnosis determined by MS-DS. Empty and full circles, negative and positive Mucorales qPCR results, respectively.

The distribution of MS-DS index values in relation to the date of IFI diagnosis and in comparison to the qPCR results is shown in Fig. 6. Among the serum samples tested before a mycological diagnosis was obtained, three (from three patients) were positive by MS-DS and six (from five patients) were positive by qPCR. An example of the kinetic evolution of MS-DS and qPCR in a patient from whom numerous samples were available is shown in Fig. 3C. A steady increase in MS-DS index values was observed during the 2 months of follow-up, whereas DNA circulation could be detected only between day 0 and day 20.

## DISCUSSION

In contrast to diagnostic tests for obligate pathogens, whose detection is indicative of disease, diagnostic tests for opportunistic pathogens have to discriminate between the presence of microbes as endo- or exosaprophytes and their shift to a pathogen (19). This is a kinetic process where the assay has to present the best compromise between specificity and sensitivity over time from disease onset and its evolution to invasive infection. Currently, for IC and IA, only two types of tests have been shown to be clinically useful for disease management (6, 21, 32, 33). On the one hand, the Platelia Candida Ag Plus test and Platelia Aspergillus Ag test, which detect Man and GM, respectively, are considered specific but have a low sensitivity (especially for the detection of Man). On the other hand, the Fungitell test, which detects BDG, is considered more sensitive but less specific. The results of our previous study concerning MS-DS and those of the current study are in agreement with these conclusions. In both studies, MS-DS appeared to provide intermediate results, being more sensitive than the Platelia tests but more specific than the test for BDG (28). It is therefore suggested that MS-DS could be useful in the management of patients at risk of IC and IA.

For some other IFI, such as those caused by Mucorales, the lack of glucans in their cell wall and negativity for BDG in the context of host invasion have led to considerable efforts to develop alternative diagnostic methods; a specific PCR is now available (24). qPCR is of great help in the diagnosis of MM, whose emergence is worrying in terms of incidence and severity (34). MS-DS was previously shown to be positive during MM (28). In the present study, a comparison of the results of MS-DS with the results of qPCR showed a similar performance, confirming that a panfungal diagnostic assay is useful in daily practice when an IFI is suspected without mycological evidence. The reproducibility of the sensitivity values in mono- and multicenter studies confirms the robust character of the Platelia and BDG tests, in line with their extensive use worldwide for several decades. A similar reproducibility and, thus, robustness were observed for MS-DS. Additional information regarding MS-DS specificity concerned the absence of false positivity associated with *Nocardia* infection, in contrast to BDG detection, as reported previously (35) and as recently observed for one of the two patients included in this study. Conversely, among the three neutropenic patients who were included as IA controls and who were revealed to have subsequently developed IA, none were positive for either GM or BDG, while MS-DS index values were positive and above the limit of significance in two patients. Although these results could be considered a coincidence, the long delay before disease development should be considered with caution due to the possible subclinical character of DS circulation. Unpublished data described the structure and function of DS and showed that among the *m/z* 365 hex-disaccharide signals (27) is trehalose, an important fungal metabolite (36). What the present study, investigating four different IFI biomarkers in comparison with DS, makes particularly obvious is the different kinetics of their circulation, as shown in Fig. 3. From a pathophysiological point of view, the metabolite DS has kinetics of synthesis and release from fungal cells different from those of cell surface-associated Man and GM or BDG, which is thought to be deeply anchored in the cell wall. These fungal polysaccharides are synthesized *in situ*, and then different processes of degradation take place as a result of either fungal or host carbohydrate catabolism. In parallel, binding to host receptors, catabolism by host soluble enzymes, and circulation as immune complexes make the levels in the circulation completely different. The efficiency of human mannosidases, naturally present for degrading human glycoproteins, and the large amount of antimannan antibodies present in IC patients are responsible for the rapid clearance of mannan. In contrast, mammals are poorly equipped for degrading glucans, which are not self-components, and their poor immunogenicity does not help with their clearance, explaining their longer persistence than mannans (37, 38). Despite considerable efforts to solve the problems of DNA extraction and standardization, the lack of knowledge regarding the relationship between clinical outcome and *Candida* PCR results has prevented clinical recommendations (39). Recent progress has been made by the association of PCR with magnetic resonance detection in the T2MR system, leading to good specificity and increased sensitivity with regard to blood cultures for IC (25, 40). For MM and in the absence of other biomarkers, qPCR represents significant progress, as demonstrated by a large collaborative study showing its ability to confirm the diagnosis. Furthermore, the survival rate was significantly higher in patients with an initially positive PCR result that became negative after treatment initiation than in patients whose PCR result remained positive (41). In our study, Mucorales qPCR and MS-DS were complementary since all serum samples were positive by at least one test. Although the significance of DS persistence should be compared to a positive PCR result, these results emphasize the benefit of MS-DS in patient care, principally due to the combination of biomarkers whose kinetics of synthesis and release differ during the pathogenic processes. The panfungal characteristics of MS-DS adapted to the broad diversity of emerging fungal pathogens presents some advantages for first-line screening. This simple, robust physicochemically based technology is easily implementable in the majority of clinical mycology laboratories now equipped with MALDI-TOF MS and can be adapted to single tests or a large series (42). However, as the present collaborative study was retrospective, its promising results have to be confirmed through a prospective study. More generally, this method, which allows the early identification and quantification of fungal glycans, is in its infancy, and studies are in progress to explore its potential. In parallel, studies concerning the contribution of MS-DS complemented with currently recommended tests are exploring the possibility of improving antifungal stewardship based on a better knowledge of the diagnostic and prognostic significance of glycobiomarkers.
